# The multi-slit very small angle neutron scattering instrument at the China Spallation Neutron Source

**DOI:** 10.1107/S1600576724000815

**Published:** 2024-03-21

**Authors:** Taisen Zuo, Zehua Han, Changli Ma, Songwen Xiao, Xiong Lin, Yuqing Li, Fangwei Wang, Yongcheng He, Zhenqiang He, Junsong Zhang, Guangyuan Wang, He Cheng

**Affiliations:** a Spallation Neutron Source Science Center, Dongguan, 523803, People’s Republic of China; b Institute of High Energy Physics (IHEP), Chinese Academy of Science (CAS), Beijing, 100049, People’s Republic of China; c School of Nuclear Science and Technology, University of Chinese Academy of Sciences, Beijing, 100049, People’s Republic of China; d Institute of Physics, Chinese Academy of Sciences, Beijing, 100190, People’s Republic of China; Oak Ridge National Laboratory, USA; North Carolina State University, USA

**Keywords:** multi-slit very small angle neutron scattering instrument, MS-VSANS, neutron focusing, spallation neutron sources, absolute intensity, polarized neutrons

## Abstract

A multi-slit very small angle neutron scattering (MS-VSANS) instrument has been finally accepted at the China Spallation Neutron Source (CSNS). It is the first spallation neutron source based VSANS instrument. MS-VSANS has a good signal-to-noise ratio and can cover a wide scattering vector magnitude range from 0.00028 to 1.4 Å^−1^. In its primary flight path, a combined curved multichannel beam bender and sections of rotary exchange drums are installed to minimize the background downstream of the instrument. An exchangeable multi-slit beam focusing system is integrated into the primary flight path, enabling access to a minimum scattering vector magnitude of 0.00028 Å^−1^. MS-VSANS has three modes, namely conventional SANS, polarizing SANS and VSANS modes. In the SANS mode, three motorized high-efficiency ^3^He tube detectors inside the detector tank cover scattering angles from 0.12 to 35° simultaneously. In the polarizing SANS mode, a double-V cavity provides highly polarized neutrons and a high-efficiency ^3^He polarization analyser allows full polarization analysis. In the VSANS mode, an innovative high-resolution gas electron multiplier detector covers scattering angles from 0.016 to 0.447°. The absolute scattering intensities of a selection of standard samples are obtained using the direct-beam technique; the effectiveness of this method is verified by testing the standard samples and comparing the results with those from a benchmark instrument. The MS-VSANS instrument is designed to be flexible and versatile and all the design goals have been achieved.

## Abstract 2

The first spallation neutron source based very small angle neutron scattering instrument has been constructed and accepted at the China Spallation Neutron Source. The instrument finds applications in multidisciplinary fields of soft matter, condensed matter physics, materials science and others.

## 1. Introduction

Small-angle neutron scattering (SANS) has been a unique structural probe in multidisciplinary research for many years, characterizing structure and interactions on length scales from 1 nm to a few hundred nanometres. Traditional SANS instruments employ the pinhole geometry to collimate the incident neutrons, *e.g* LOQ (Heenan *et al.*, 1997 ▸) (ISIS, UK) and 8m SANS (Glinka *et al.*, 1998 ▸) (National Bureau of Standards/National Institute of Standards and Technology, NBS/NIST, USA). Restricted by the large beam cross section and the flux of incident neutrons, the minimum scattering vector magnitude *Q* [*Q* = 4πsin(θ/2)/λ, where θ is the scattering angle and λ is the neutron wavelength] accessible for traditional pinhole SANS cannot usually be lower than 0.003 A^−1^. The use of an MgF_2_ lens in a pinhole system successfully reduces the minimum *Q* to 0.001 Å^−1^, which means the size of the scattering objects cannot be larger than 300 nm (Zhang *et al.*, 2014 ▸; Glinka *et al.*, 1998 ▸). However, various materials have characteristic length scales exceeding this size, *e.g.* porous materials (Kampman *et al.*, 2016 ▸), multi-scale fractal materials (Zhou *et al.*, 2016 ▸), biological macromolecule aggregates (Sauter *et al.*, 2016 ▸) or crude oil (Headen *et al.*, 2009 ▸), demanding an upgrade of the instruments.

To extend the accessibility to smaller *Q* values, new focusing techniques need to be implemented. A SANS instrument equipped with new focusing techniques and high-resolution detectors to push the minimum *Q* below 0.001 Å^−1^ is called a very small angle neutron scattering (VSANS) instrument (Barker *et al.*, 2022 ▸). To access smaller *Q* below 0.001 Å^−1^, much effort has been made and progress has been achieved: for example, an extremely long instrument with long-wavelength neutrons on D11 at the ILL (Grenoble, France) Lindner & Schweins, 2010 ▸), effective application of MgF_2_ and magnetic lenses on SANS-J-II at the Japan Research Reactor (Koizumi *et al.*, 2006 ▸) and successful application of a toroidal reflection mirror on KWS-3 at the Jülich Centre for Neutron Science in Germany (Radulescu *et al.*, 2005 ▸). Some other VSANS instruments using the lens approach are still under development. Oku and co-workers have tried to use magnetic lenses to focus neutrons (Oku *et al.*, 2007 ▸; Yamada *et al.*, 2015 ▸). Yamada *et al.* (2011 ▸, 2015 ▸) and Zuo *et al.* (2022 ▸) are developing a modulating permanent-magnet sextupole for a time-of-flight (TOF) SANS instrument at a spallation neutron source. Liu and co-workers have worked on a toroidal Wolter mirror to focus neutrons (Guo *et al.*, 2014 ▸; Liu *et al.*, 2013 ▸).

The most feasible technique for focusing a white neutron beam at a spallation source up to now has been the use of multi-slit collimators (Zuo *et al.*, 2016 ▸). The concepts of multichannel collimator converging diaphragms or Soller collimators to upgrade traditional SANS instruments were proposed in the 1970s by Nunes, Carpenter and co-workers (Nunes, 1974 ▸; Carpenter & Faber, 1978 ▸). The first testing of focusing neutrons with multi-pinhole apertures was at NIST by Glinka *et al.* (1986 ▸). Crossed converging Soller collimators were successfully installed at the Intense Pulsed Neutron Source (IPNS, Argonne, Illinois, USA) in 1997 (Thiyagarajan *et al.*, 1997 ▸). Other kinds of multichannel collimator techniques have also been introduced thereafter (Margaça *et al.*, 2000 ▸; Falcao *et al.*, 2002 ▸; Falcão *et al.*, 2003 ▸; Len *et al.*, 2004 ▸). In 2006, a compact VSANS instrument with 4 m long collimation using both multi-slit and multi-pinhole apertures was built by Désert *et al.* (2007 ▸) at LLB, France. With the multi-pinhole approach, the minimum *Q* is 2 × 10^−4^ Å^−1^. In 2006, Helmholtz-Zentrum Berlin (HZB), Germany, started to build a new TOF small-angle scattering instrument V16/VSANS (Vogtt *et al.*, 2014 ▸). In 2009, Barker and co-workers began to build a new VSANS instrument with both multi-slit and multi-pinhole options at NIST (Barker *et al.*, 2022 ▸). In 2014, Jaksch and co-workers proposed a new VSANS instrument (Jaksch *et al.*, 2014 ▸) with a multi-slit option for the European Spallation Source (ESS, Lund, Sweden).

Recently, with a novel design of combined translational and rotational exchange of optical components and a laser-aided alignment of the multi-slit system (Luo *et al.*, 2021 ▸), the first VSANS instrument based on a spallation neutron source has been constructed at the China Spallation Neutron Source (CSNS). With twelve multi-slits to converge the multiple narrow beams to the surface of a high-resolution gas electron multiplier (GEM) detector, a minimum *Q* of 0.00028 Å^−1^ can be achieved. A variable collimation length offers a flexible trade-off between neutron flux and resolution. Three consecutive movable detectors (roughly 1 m^2^ effective area each) cover scattering angles from 0.12 to 35° simultaneously. Polarized neutrons are available with a double-V polarizing cavity. Standard samples have been used for the calibration of the instrument and a direct-beam technique was employed to obtain the absolute intensity.

The multi-slit very small angle neutron scattering (MS-VSANS) instrument is financially supported by the Department of Science and Technology of Guangdong Province. Development of the instrument began in December 2019 and the first neutron measurement was made in January 2023.

In this paper, we first present a general description of the VSANS instrument. This is followed by a description of the design and performance of individual components of the instrument such as the beam bender, chopper, collimation, polarizing and detector systems. Finally, the results of standard sample measurements are reported.

## 2. Instrumentation

### 2.1. Instrument geometry

**Figure 1:**
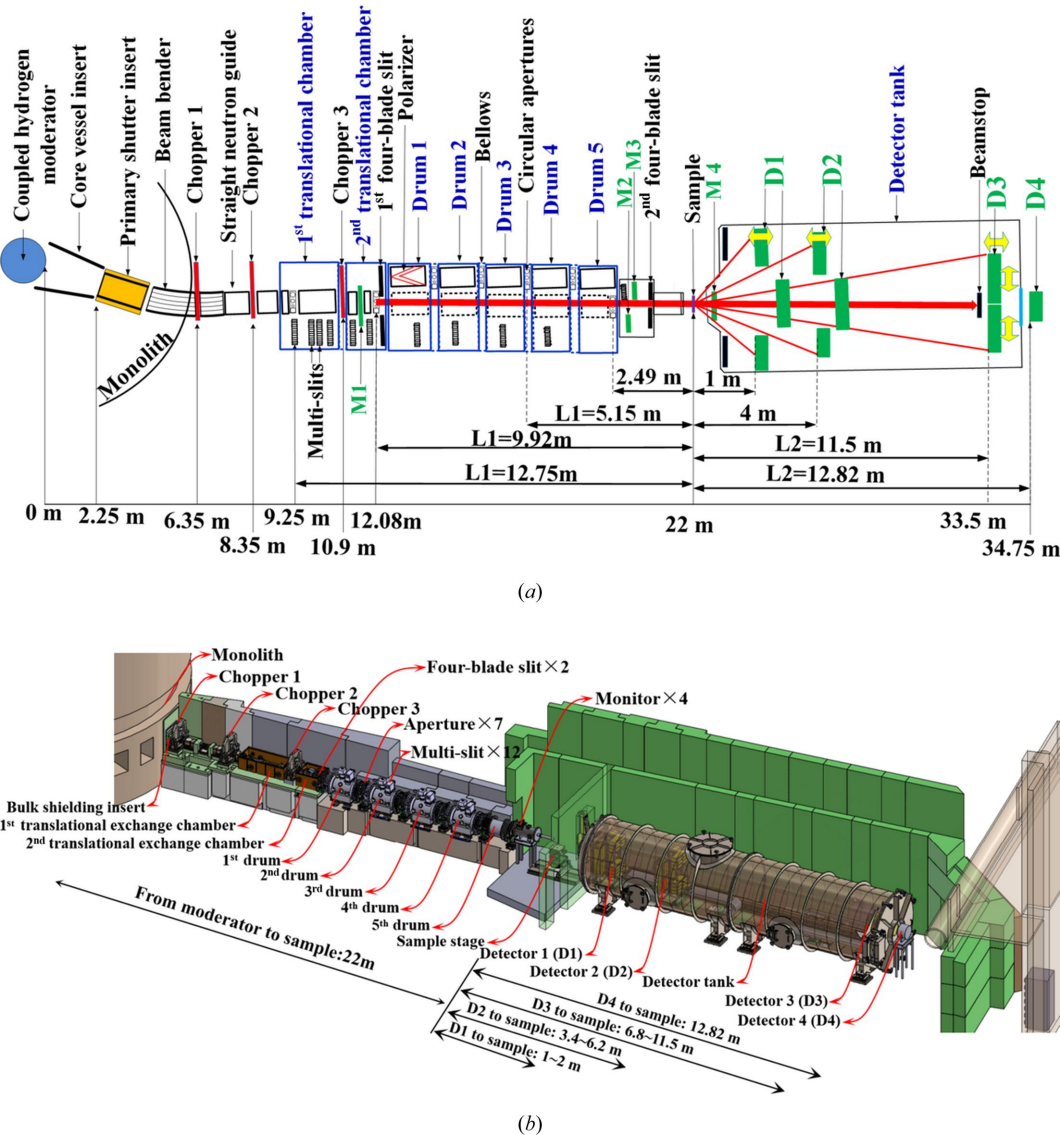
(*a*) A schematic diagram of the VSANS instrument. (*b*) An overall mechanical drawing of the instrument.

The VSANS instrument is located on beamline 14 of the target station of CSNS (Wang *et al.*, 2013 ▸). CSNS is currently operating at 140 kW with a proton pulse repetition rate of 25 Hz. The coupled hydrogen moderator of CSNS is selected to provide high-flux cold and thermal neutrons. The blueprint of the instrument and overall mechanical drawing are shown in Fig. 1 ▸. Defining the surface of the moderator as the origin, the total length of the instrument (from the origin to the last detector) is 34.75 m. A longer instrument means lower background and more flexible and lower minimum *Q*, but also means a narrower waveband (Δλ ∝ 1/*L*, where *L* is the distance from the moderator to the detector). This problem of a narrow waveband can be compensated for by wide detector coverage and a frame-skipping mode of the choppers, which will be introduced below. The maximum sample sizes of the SANS and VSANS modes were designed as ø15 mm and 15 × 30 mm, inferring an optimal inner cross section of all the neutron guides of 30 × 40 mm (Mildner & Carpenter, 1984 ▸). No focusing or tapering neutron guides were applied because of the limited gain in flux at the sample as calculated by Monte Carlo simulations.

**Figure 2:**
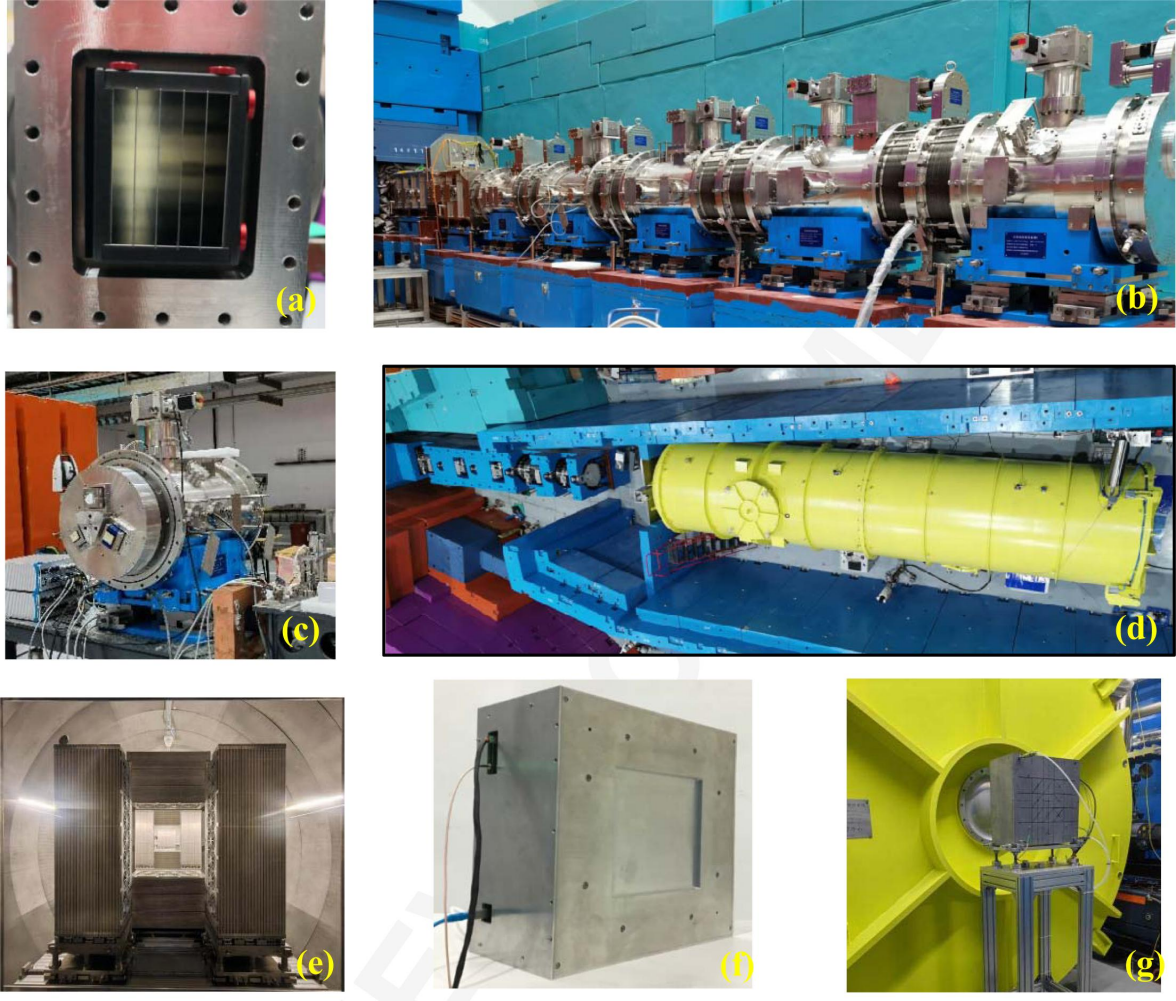
(*a*) The neutron beam bender in the bulk shielding insert. (*b*) The rotary exchange drums after installation. (*c*) The first rotary exchange drum. (*d*) The detector tank. (*e*) The three ^3^He detectors, viewed from the sample site. (*f*) The high-resolution GEM detector D4. (*g*) D4 installed after the detector tank.

As shown in Fig. 1 ▸, the first section of straight neutron guide was installed inside a 2 m long primary shutter insert (PSI) which is 2.25 m away from the moderator. The second section is a 2.095 m long beam bender [Fig. 2 ▸(*a*)] installed inside a bulk shielding insert (BSI). After the first section of the beam bender, there is the first disc chopper (Chopper 1) located at 6.35 m, followed by another 1.00 m long beam bender. The two sections of beam benders change the direction of the beam by up to 1.91°. After the bender there is a section of straight guides to homogenize the neutron beam, followed by the second disc chopper (Chopper 2) located at 8.35 m. The third chopper (Chopper 3) is located at 10.90 m between the two translational exchange chambers.

The collimation system of the VSANS instrument [Fig. 1 ▸(*a*)] consists of circular apertures, multi-slit apertures, four-blade slits, flight tubes and neutron guides. All these components of the collimation system are sealed inside two translational exchange chambers (Chamber 1 and Chamber 2), five rotary exchange chambers [drums 1 to 5, Figs. 2 ▸(*b*) and 2 ▸(*c*)] and one small translational exchange chamber before the sample (Fig. 1 ▸). All these chambers share the same vacuum and are connected with bellows.

After the collimation system, there is a sample stage with rails to move forward and backward to an extent of 1 m. The usual sample position from the moderator is about 22 m. After the sample, three movable ^3^He position-sensitive detectors (Detectors 1 to 3 or D1, D2, D3) are located inside the detector tank [Fig. 2 ▸(*d*)] with usual sample-to-detector distances (SDDs) of 1, 4 and 11.5 m, respectively [Fig. 2 ▸(*e*) and Fig. 1 ▸]. The high-resolution GEM detector [Figs. 2 ▸(*f*) and 2 ▸(*g*)] is located 12.82 m from the sample outside the detector tank.

The geometry of the three ^3^He detectors viewed from the sample position is shown in Fig. 2 ▸(*e*). A wide scattering angle range from 0.12 to 35.26° can be covered simultaneously. On the one hand, this is more efficient for instruments with a narrow wavelength band; on the other hand, dynamically changing samples can be measured with a single setup. For example, D33 (Dewhurst, 2008 ▸) at ILL in France, SANS2D (Heenan *et al.*, 2011 ▸) at ISIS, UK, and Bilby (Sokolova *et al.*, 2019 ▸) at ANSTO in Australia use a rear and a front detector for the collection of scattered neutrons. The NIST VSANS instrument employs three movable detectors to cover scattering angles from 8 × 10^–5^ to π/6 rad (30°) simultaneously (Barker *et al.*, 2022 ▸). TAIKAN at J-PARC in Japan can simultaneously cover a *Q* range from 0.005 to 17 Å^−1^ by applying multiple fixed detector banks (Iwase *et al.*, 2018 ▸). There are four banks for D1 and D2, and they are left–right banks (400 × 1000 mm) and top–bottom banks (400 × 600 mm). The position of the detector is defined by the position of the left–right banks, since the top–bottom banks are 0.43 m behind the left–right banks. Detector 3 only has left–right banks (500 × 1000 mm) which can move apart to make sure that D4 [Figs. 2 ▸(*f*) and 2 ▸(*g*), effective area 200 × 200 mm] can see the neutrons. There are five beamstops (with diameters of 50, 70, 90, 140 and 230 mm, respectively) in front of D3 to stop the direct beam in SANS experiments.

### 2.2. Components of the instrument

Key components of the instrument include straight neutron guides, a beam bender, three choppers, two translational exchange chambers, five rotary drums, a polarizer, multi-slit apertures, sample stages, sample environments and detectors. We will start by introducing the designs and functions of these components.

**Figure 3:**
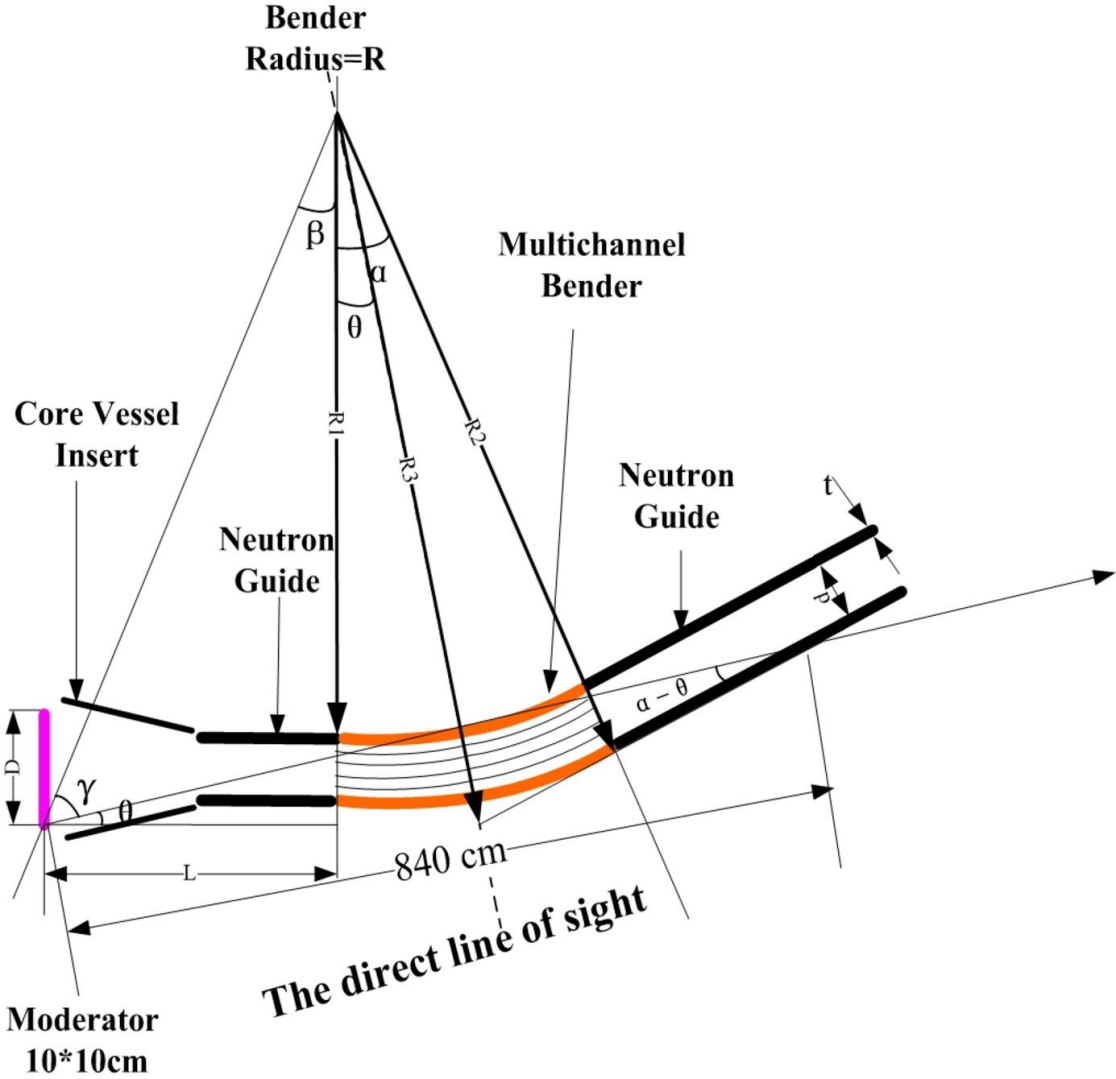
A schematic view of the direct line of sight.

In a SANS instrument, straight neutron guides propagate neutrons straight to the front of the source aperture. A beam bender is a curved neutron guide with several channels to divert neutrons from the moderator’s direct line of sight at the instrument’s downstream end. In the conceptual design of the instrument, the parameters of the bender were optimized to be 3 m long, five channels and 90 m radius of curvature. The bender’s coating is *m*3.6 on the concave side and *m*2 Ni/Ti supermirror on the convex and top sides. The neutron wavelength cutoff of the bender is 1.85 Å by formula (1) (Schmidt *et al.*, 1996 ▸),

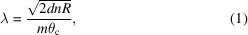

where *d* is the guide width, *n* is the number of channels, *R* is the radius of curvature, *m* is the *m*-value of the supermirror and θ_c_ is the total reflection angle of natural nickel which is 0.00173 rad Å^−1^. The neutron guides before the beam bender are made of float glass, while the neutron guides after the beam bender are made of boron float glass (4 wt% boron). All of the supermirror parts were acquired from SwissNeutronics Inc. (https://www.swissneutronics.ch/). When the reflection angle is less than the critical angle θ_c_, the reflectivity for all the supermirror coatings is close to 100%. It falls to no less than 84% at 3.6θ_c_ for the *m*3.6 supermirrors and no less than 93% at 2θ_c_ for the *m*2 supermirrors. The length of the direct line of sight is 8.36 m when the bender’s optimal settings are used, as determined by formula (2) and the Fig. 3 ▸ parameter values. After 8.36 m, blockages (sections of stainless steel and boron aluminium alloy) were put around the neutron guides to make up for the translational exchange chamber’s inadequate shielding from the chopper pits and gaps. The rotary drums further stop the direct line of sight neutrons from the moderator.




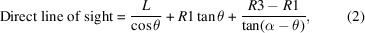

where

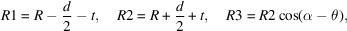




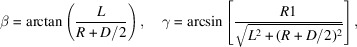




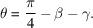




**Figure 4:**
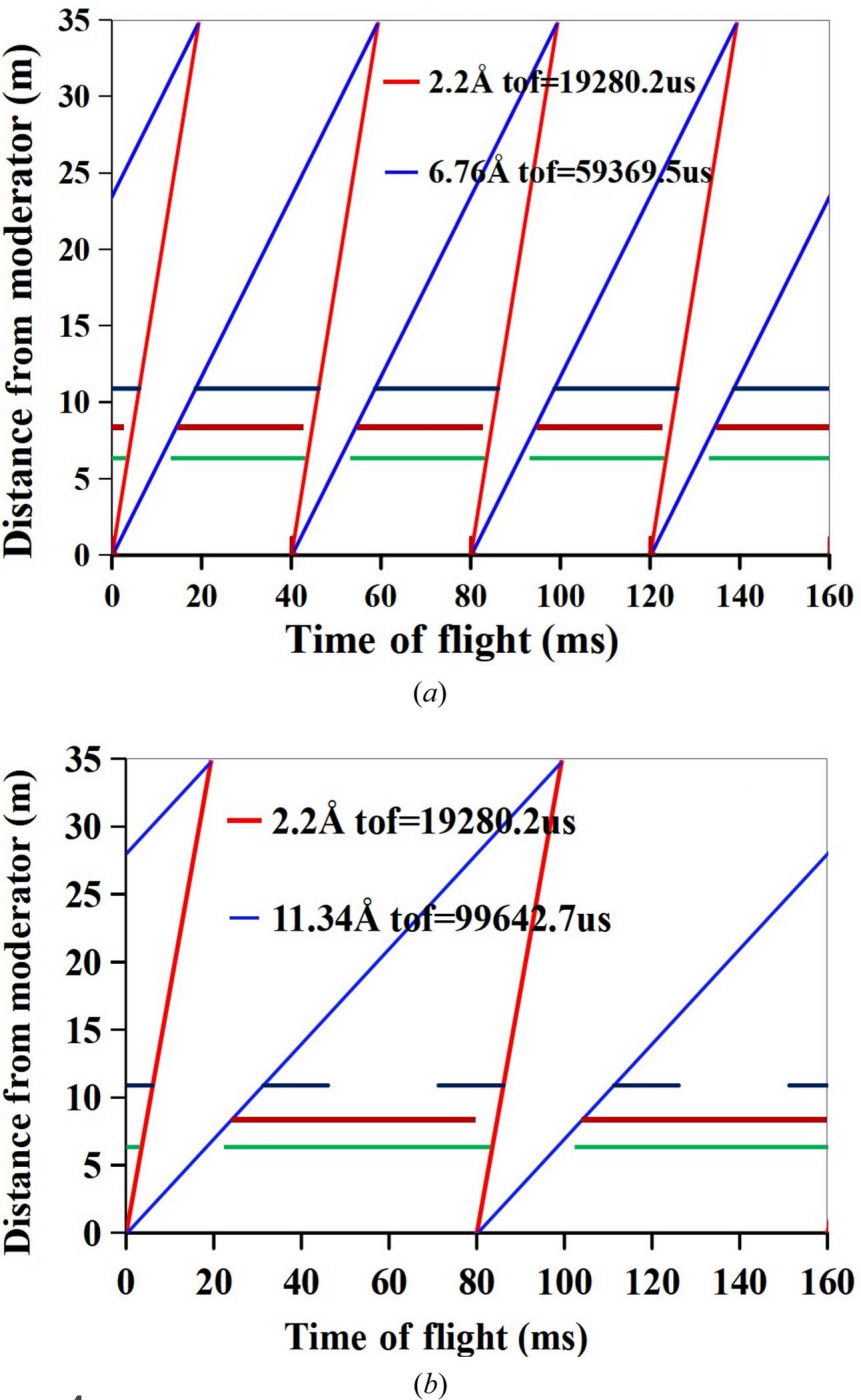
Time–distance diagrams of the chopper system, (*a*) standard mode (2.2–6.7 Å) and (*b*) frame-skipping mode (2.2–11.3 Å).

In the spaces between the bender and neutron guides, three disc choppers, designated T1, T2 and T3, were inserted. The disc choppers are neutron-absorbing discs with a fan-like form that are used to choose the neutron waveband in a pulse. The system can operate in standard mode or frame-skipping mode, by adjusting the frequency and phase of the three choppers (Zhao *et al.*, 2010 ▸). In Fig. 4 ▸, time–distance diagrams for these two modes are displayed.

As seen in Fig. 4 ▸, the standard mode or frame-skipping mode can be used to pick a wavelength bandwidth of 4.5 Å (2.2–6.7 Å) or 9.1 Å (2.2–11.3 Å). The frame-skipping mode (it removes one pulse out of every two pulses to obtain a double bandwidth) is helpful for samples that need a large *Q* coverage in one measurement or the measurement of dynamic change in the system. Table 1 ▸ displays the three choppers’ initial phase, running frequency and blade cutting angles.

Following the choppers, a collimation system was installed to switch between SANS and VSANS modes as well as between various SANS collimation lengths. These collimation parts need to be contained in chambers. Two translational exchange chambers and five rotary drums are combined because the latter have tight shielding and the former are simple to maintain and can accommodate complex devices. Two sections of neutron guides, four multi-slit apertures, one conventional circular aperture and a laser reflection mirror may all be found inside the first translational exchange chamber. For the laser alignment of the twelve multi-slit apertures (Luo *et al.*, 2021 ▸), the reflection mirror reflects a parallel laser of 405 nm wavelength from the left-hand side of the chamber to the sample point of the instrument. All these parts are freely movable in and out of the beam horizontally. However, it is not possible for the multi-slits and neutron guides to enter the beam at the same time. Aperture sizes of 31 × 41 mm, ø30 mm, ø20 mm and ø10 mm are available for the conventional aperture. Two sections of neutron guides, two multi-slit apertures, one beam monitor, one conventional aperture (optional size of 31 × 41 mm, ø30 mm, ø25 mm, ø20 mm and ø16 mm) and one four-blade slit are all present in the second translational exchange chamber. All these parts can move into or out of the beam just like they can in the first translational chamber, but the multi-slits and the neutron guides cannot move into the beam at the same time. To prevent a collision between the two, tight limits and interlocking logic have been introduced to the management of the movement of the neutron guides and multi-slits.

There are then the five sections of rotational exchange drums (each about 1.5 m long, with the exception of the final section, which is 1 m long), each followed by a conventional aperture with rotational exchange. With a spring device to eliminate backlash, the rotational accuracy of the drums has reached 0.0005° to ensure exact alignment of the neutron guides. The rotary drums have at least two optional functioning positions, the neutron guide or the flight tube. The flight tube is a rectangular segment of tube with a rough inner surface constructed of BAl alloy (30 wt% boron carbide B_4_C). To prevent reflection, fixed absorption apertures with an inner size of 31 × 41 mm are inserted roughly every 250 mm inside the flight tubes. As shown in Fig. 2 ▸(*c*), the first rotary drum also features a double-V polarizer option, which will be discussed in the following paragraph. To maintain a magnetic field stronger than 50 gauss even at the gaps, permanent magnet bars (N38 NdFeB) are put on the sides of the flight tubes, covered with iron plate yokes on the top and bottom. The rotary drums are made of stainless steel (SUS304) with a low magnetic susceptibility. A multi-slit drop mechanism on top of the first four rotary drums allows the multi-slits to be inserted into the beam only when the drums are in the flight tube positions. Conventional apertures after each drum can serve as source aperture or intermediate aperture with varying collimation lengths. After the final rotary drum, a small chamber contains exchangeable parts, including a multi-slit aperture, two beam monitors, a four-blade slit and a laser reflection mirror for the alignment of the sample environments.

**Figure 5:**
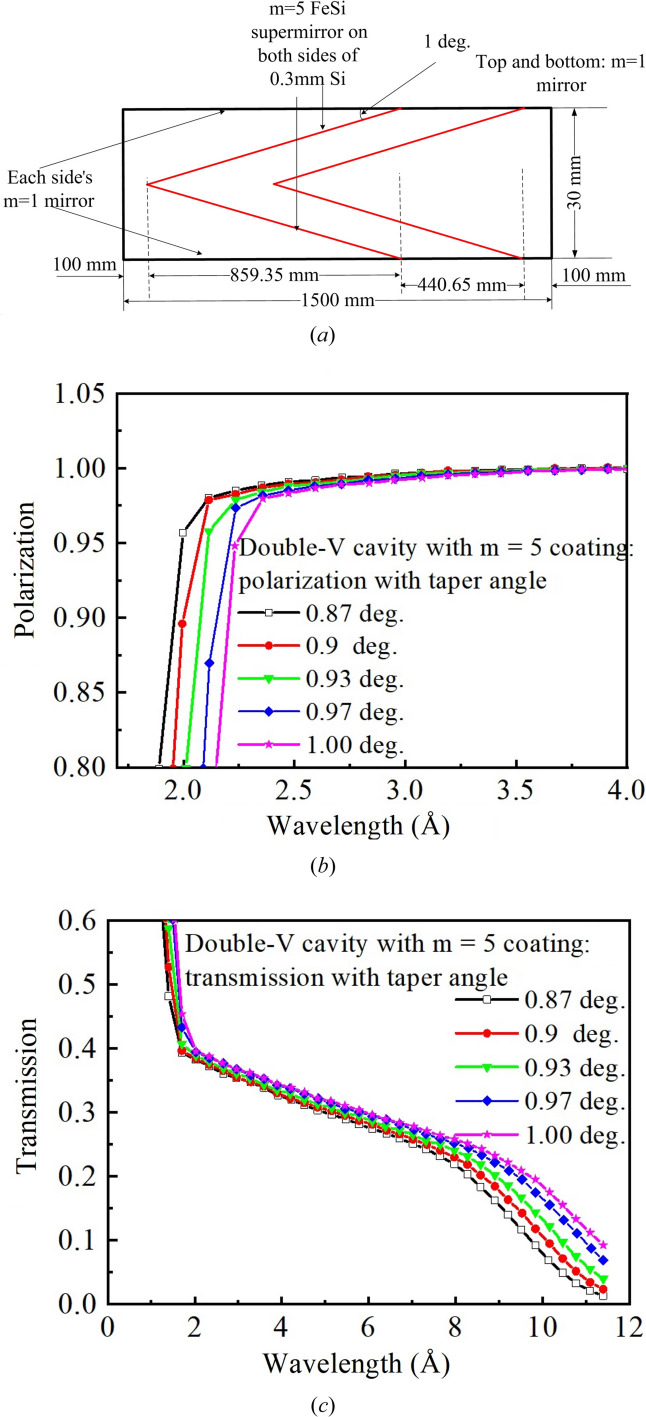
(*a*) The geometry of the double-V cavity polarizer. (*b*) The polarizability of the double-V cavity with different tilt angles of the polarizing mirror. (*c*) The transmission of the double-V cavity with different tilt angles of the polarizing mirror.

As previously mentioned, there is a polarizer inside the first rotary drum [Fig. 2 ▸(*c*)], designed to polarize cold neutrons from 2.2 to 11 Å. Fig. 5 ▸(*a*) displays the physical design of the double-V cavity polarizer. In order to create a double-V cavity, four plates of *m*5 Fe/Si supermirror with approximately 859.35 mm length and 1° tilt angle are put into the 1.5 m guide. The 1.5 m guide has been coated with *m*1 mirror. Monte Carlo simulations by SwissNeutronics Inc. show that the polarization of a double-V cavity at 2.2 Å (higher than 90%) is higher than that of a single-V cavity (about 80%) (not shown here). By contrasting the polarizability and transmission of the polarizer, as illustrated in Figs. 5 ▸(*b*) and 5 ▸(*c*), the tilt angle of the inserted mirror is optimized. For better transmission of cold neutrons from 6 to 11 Å and approximately 94% polarizability at 2.2 Å, a tilt angle of 1° is used.

**Figure 6:**
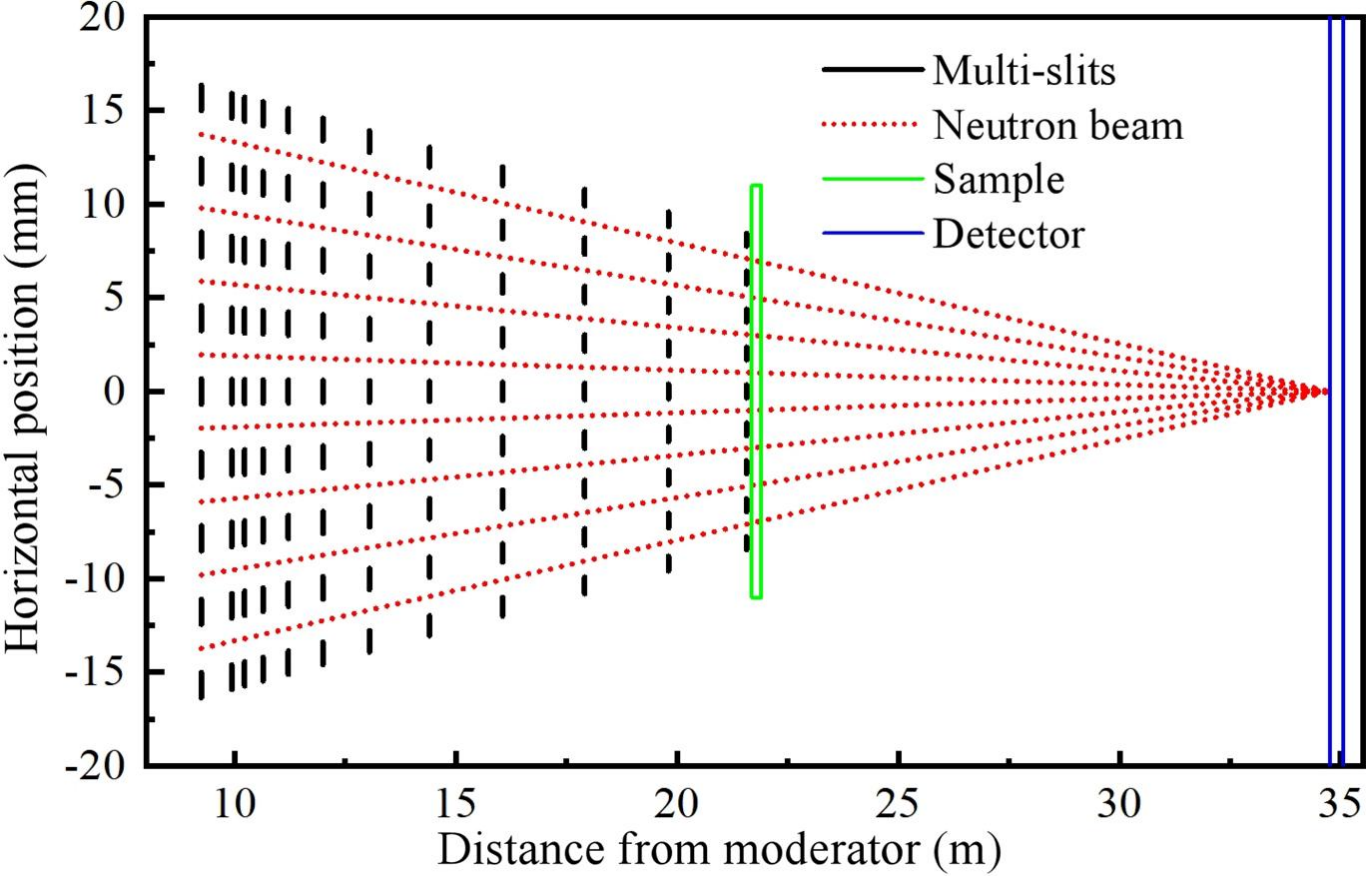
A diagram of the arrangement and focusing of the twelve multi-slits.

Twelve multi-slit apertures are integrated into the two translational exchange chambers and five rotational exchange drums of the instrument, four within the first translational chamber, two in the second translational chamber, four on the top of the rotary drum, one in the small chamber before the sample and one just before the sample. Table 2 ▸ gives specific parameters of the multi-slits. Eight narrow beams converge from the neutron guide exit at 9.25 m toward the detector surface at 34.82 m (Fig. 6 ▸). Each beam’s cross section tapers linearly from 2.55 × 40 mm at the guide exit to 1.32 × 40 mm before the sample. In this study, the arrangement of the intermediate apertures is designed using a modified version of Barker’s algorithm (https://www.nist.gov/system/files/documents/2023/04/14/the_sans_toolbox.pdf) to prevent crosstalk between adjacent beams. Reflection and scattering from the edges of the multi-slits may result in severe noise, especially close to *Q*_min_. A bevelled multi-slit structure is suggested (Wang *et al.*, 2018 ▸) with a cutting angle of 5° to alleviate this issue.

With all of the neutron optics now described, formula (3) can be used to estimate the lowest *Q* of the SANS mode and VSANS mode without taking the resolution of the detector into account:

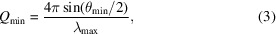

where 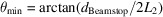 is the minimum scattering angle. In the SANS mode, the minimum *Q* can be determined to be 0.0013 Å^−1^ when the source-to-sample distance (SSD) is 12.75 m, the diameter of the beamstop *d*_Beamstop_ is 50 mm, *L*_2_ is 11.5 m and the maximum neutron wavelength λ_max_ is 10.5 Å. In the VSANS mode, with an SSD of 12.75 m, *d*_Beamstop_ of 7 mm, *L*_2_ of 12.82 m and λ_max_ of 10.5 Å, the lowest *Q* can be determined to be 0.00016 Å^−1^. Given the detector resolution and background around the beamstop, the minimum *Q* would really be larger.

Calculating the maximum *Q* in the SANS mode and VSANS mode is possible using formula (4): 

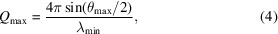

where 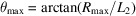 is the maximum scattering angle. In the SANS mode, when the maximum radius of the detector *R*_max_ = 0.7 m, *L*_2_ = 1 m, θ_max_ = 35° and the minimum neutron wavelength λ_min_ = 2.2 Å, the maximum *Q* can be calculated to be 1.72 Å^−1^. In the VSANS mode, when *R*_max_ = 0.1 m, *L*_2_ = 12.82 m, θ_max_ = 45° and λ_min_ = 2.2 Å, the maximum *Q* can be determined to be 0.022 Å^−1^.

**Figure 7:**
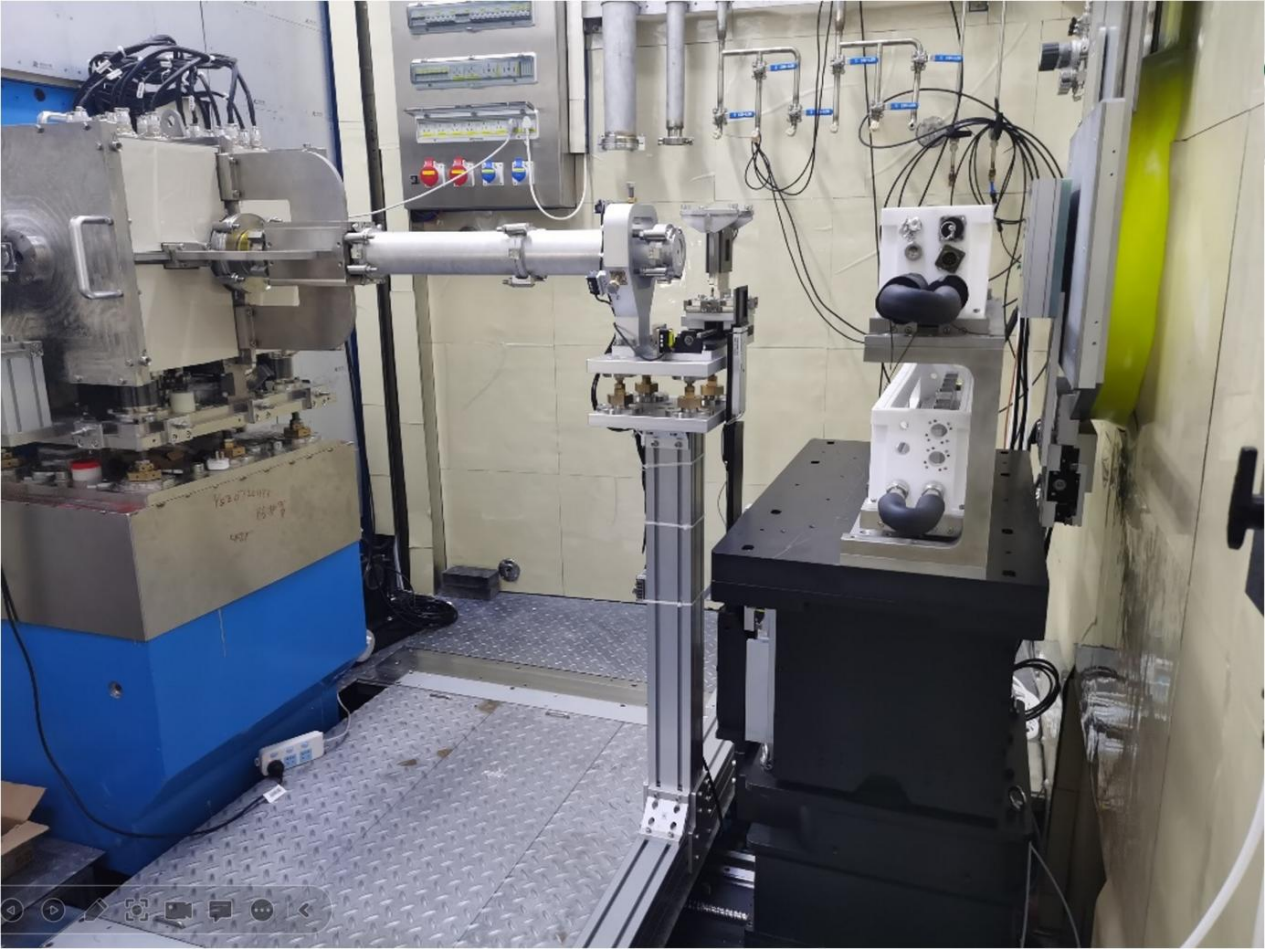
The sample stages and sample changer in the sample hutch of the VSANS instrument.

In the sample hutch (Fig. 7 ▸), two stacked sample stages have been designed to accommodate various sample environments. A heavy bearing stage with 1000 kg bearing is at the bottom and a light bearing stage with 150 kg bearing is at the top. To accommodate diverse sample environments, the heavy bearing stage is mounted on two tracks and allowed to travel forward and backward over 1.0 m. There is currently a double-decker sample changer on the top of the light bearing stage equipped with a cooling liquid bath (−30 to 110°C) and a heater (room temperature to 300°C) (Hu *et al.*, 2023*a* ▸). When measuring with banjo-type cells, 20 samples may be loaded into the sample changer at once. In the VSANS mode, 16 samples can be loaded at once with rectangular cells.

For the measurement of magnetic samples, a 5 T superconductive magnet can be installed on the heavy bearing stage. The majority of the sample stages are made of aluminium alloy and the front of the detector tank is made of stainless steels to avoid the magnetic force of the stray field. Full polarizing scattering is also feasible, although the radio frequency polarizing flipper and the *in situ* SEOP ^3^He polarizing analyser (Huang *et al.*, 2021 ▸; Zhang *et al.*, 2022 ▸) may be affected by stray field from the 5 T magnet, restricting the maximum magnetic field to less than 2 T.

From the French business Xenocs (https://www.xenocs.com/saxs-products/) we purchased a Nano-inXider small-angle X-ray scattering (SAXS) instrument. With the vertical geometry of the Nano-inXider, simultaneous SANS and SAXS experiments are possible by lifting the Xenocs SAXS instrument onto the sample position of the VSANS instrument. In this setup, the neutron and X-ray beams are perpendicular to each other and both maintain a 45° angle with the sample plane (Metwalli *et al.*, 2020 ▸).

Further developments include a furnace (Hu *et al.*, 2023*b* ▸), temperature jump, rheometer, stop-flow, cryostat *etc.* The sample environment group of CSNS is willing to create customized sample environments upon request from the user.

All the detectors are provided by the detector group of CSNS. The efficiency and resolution of the three ^3^He detectors have been calibrated to be 82.2% (±3.2%) at 2 Å and 8 mm (FWHM), respectively (Jiang *et al.*, 2022 ▸). Alignment of the pixels between different tubes of the detector has been done with a mask on beamline 20 of CSNS. A laser tracker has been used to align and survey the position of the entire detector and the tubes relative to the sample. The high-resolution detector (D4 210 × 210 mm with 256 × 256 pixels and a pixel size of 0.82 × 0.82 mm) is a boron-plated GEM (Sauli, 1997 ▸) detector developed by the detector group of CSNS (Zhou *et al.*, 2020*a* ▸). The efficiency and resolution have been calibrated to be 24% (±2%) at 6 Å and 2.0 mm (FWHM), respectively. Two types of beam monitors were installed to measure the beam profile and transmission of the sample. One is the 4562N type monitors with an effective area of 5.1 × 6.4 cm purchased from ORDELA Inc. (https://ordela.com/). The other is a ceramic GEM-based neutron beam monitor developed by the detector group of CSNS (Zhou *et al.*, 2020*b* ▸).

## 3. Methods

### 3.1. Absolute intensity calibration

Absolute intensity calibration means obtaining the differential scattering cross section of a sample which is free from the parameters of the instrument. It is defined as the ratio of the number of neutrons scattered per second into unit solid angle (neutrons s^−1^) divided by the incident neutron flux (neutron cm^−2^ s^−1^) and thus has the dimensions of area (cm^2^) [dσ(*Q*)/dΩ; Wignall & Bates, 1987 ▸]. On normalizing to unit sample volume it is called the macro differential cross section [dΣ(*Q*)/dΩ] with units of cm^–1^. Calibration of the absolute intensity or absolute calibration is essential to both SANS and VSANS instruments because it is necessary for the determination of molecular weight, number density, volume fraction, specific surface area *etc.* of the scatterers.

There are two approaches to obtaining the macro differential cross section. The first one is the direct-beam technique, and we can use a direct incident beam to normalize the scattered intensity. The second one involves employing standard samples with known absolute scattering intensity at zero scattering angle from theoretical calculations. Here, we use the first method to get the absolute intensity and then compare it with that from a benchmark instrument and theoretical calculations.

Five measurements are needed for the direct-beam technique: (i) a direct-beam measurement *I*_direct_*n*_ (*n* denotes the lambda bin) with a small sample aperture and nothing at the sample site; (ii) a direct-beam measurement with a small sample aperture and a sample in the cell *I*_S-C_direct_*n*_; (iii) a direct-beam measurement with an empty cell and a small sample aperture *I*_C_direct_*n*_; (iv) a scattering measurement with a sample in the cell and a large sample aperture *C*_S-C_*i*,*j*,*n*_ (*i* and *j* denote the detector pixel IDs in the *X* and *Y* direction, respectively); (v) a scattering measurement with an empty cell and a large sample aperture *C*_C_*i*,*j*,*n*_. With known sample thickness *d*_sample_, the absolute intensity of the sample can be expressed as

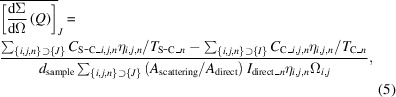

where

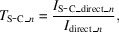




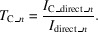




*A*_direct_ is the area of the small sample aperture in the direct-beam measurement and *A*_scattering_ is the area of the large sample aperture in the scattering measurements. 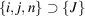 means mapping the counts in the (*i*, *j*, *n*) matrix to the radial *Q* space based on *Q* = 4πsin(θ/2)/λ. η_*i*,*j*,*n*_ is the efficiency of the detector pixels. We assume that the efficiency of every pixel is the same, so the η_*i*,*j*,*n*_ in the numerator and denominator cancel out. The same is true for η_*i*,*n*_ in equation (6).

When it comes to the absolute intensity of the VSANS mode, the same formula still applies, but *Q* should be replaced by *Q*_*X*_ [*Q*_*X*_ = 4πsin(θ_*X*_/2)/λ, where θ_*X*_ is the scattering angle in the horizontal direction]. Since the GEM detector can accept the direct beam without saturation, a small sample aperture is not needed. The absolute scattering intensity can be expressed as follows:

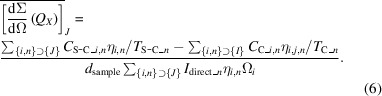




### 3.2. Experiments and samples

SANS experiments were carried out with an SSD of 12.75 m, a 30 mm source aperture and a 6 mm sample aperture. Three standard samples were measured:

(i) A mixture of polystyrene and deuterated polystyrene (PS/d-PS) as a tablet (ø18 × 1.5 mm) from the National Institute of Standards and Technology (NIST) with a mol­ecular weight of about about 200 kg mol^−1^.

(ii) Fully deuterated polyethyl­ene glycol (PEG-d4) as a tablet (ø18 × 2 mm) with a molecular weight of 2400 g mol^−1^.

(iii) Glassy carbon (SRM 3600) as a tablet (10 × 10 × 1 mm) purchased from NIST.

VSANS experiments were carried out with two standard samples, 540 and 195 nm diameter PS spheres in DHO (an equal mixture of H_2_O and D_2_O) at 1 wt% concentration. No further modifications were made or surface decorations done on the two samples. Scanning electron microscopy (SEM) experiments showed that the standard deviation of the diameter of the spheres is less than ±3.5%. The PS spheres were purchased from Suzhou Knowledge & Benefit Sphere Tech. Co. Ltd of China (https://www.kbspheres.com/).

## 4. Initial experimental data

Three standard samples were selected for calibration of the absolute scattering intensity of the SANS mode of the instrument, *i.e.* PS/d-PS, PEG-d4 and glassy carbon, as described in Section 3.2, *Experiments and samples*. Five measurements were carried out on each sample with a collimation length of 12.75 m: (i) direct-beam measurement with nothing at the sample site, (ii) direct-beam measurement with a sample in the cell, (iii) direct-beam measurement with only the cell, (iv) scattering measurement with a sample in the cell and (v) scattering measurement with only the cell. Direct-beam measurement means a 2 or 1 mm sample aperture with no beamstop, and both direct beam and scattered beam are recorded by the detector. Scattering measurement means a 6 mm sample aperture with a beamstop to block the direct beam. Data from the three samples were reduced with formula (5) to get the scattering profile and absolute scattering intensity.

**Figure 8:**
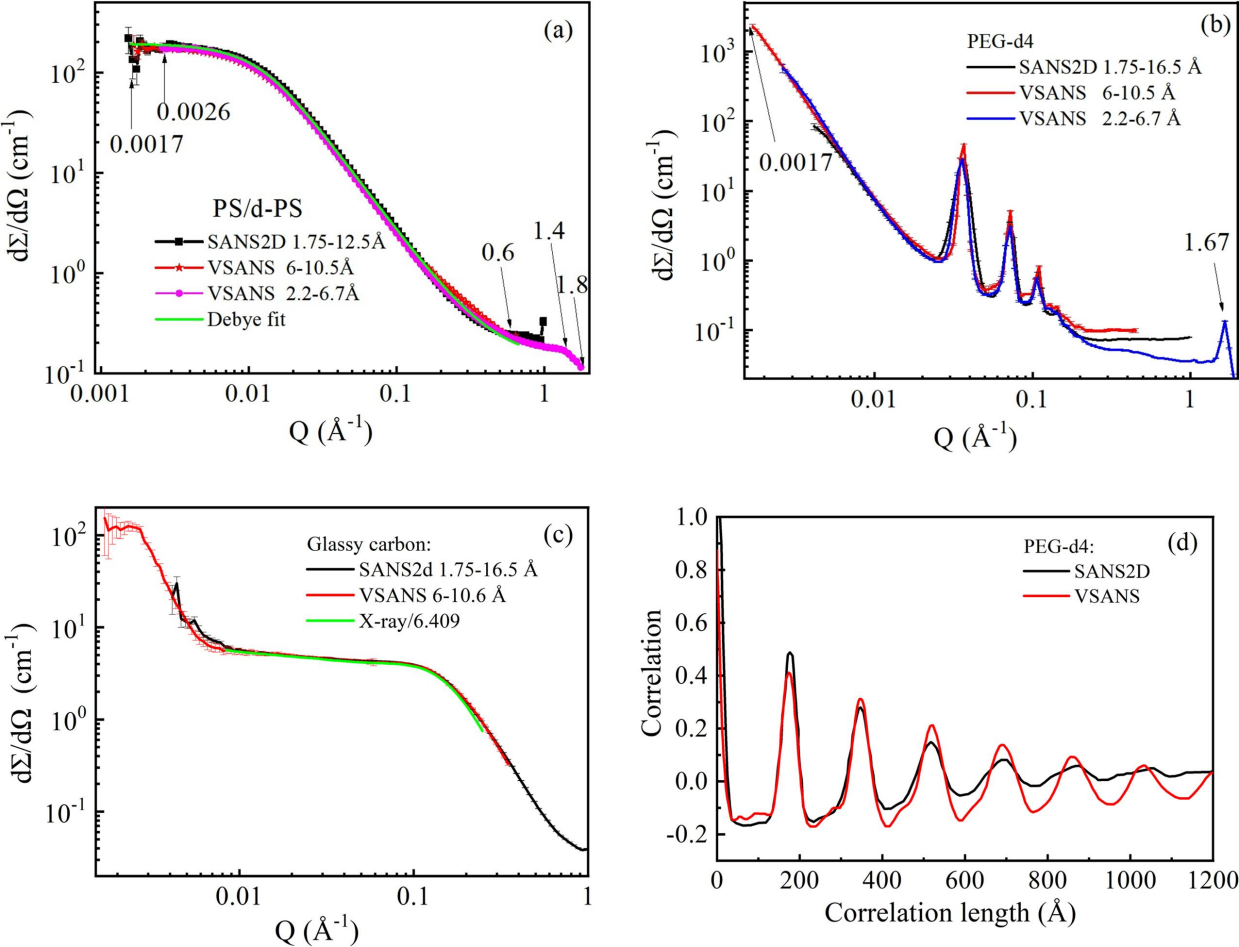
Data from the SANS mode of the CSNS VSANS instrument compared with SANS2D: (*a*) PS/d-PS, (*b*) PEG-d4 and (*c*) glassy carbon. (*d*) The correlation functions of the PEG-d4 scattering profiles using *SASView*.

The reduced scattering profiles of the three standard samples match very well with the data from the benchmark instrument SANS2D, as shown in Fig. 8 ▸. The CSNS-VSANS and SANS2D data for PS/d-PS almost overlap with each other [Fig. 8 ▸(*a*)]. Credible *Q* ranges for the 6–10.5 Å and 2.2–6.7 Å data are 0.0017–0.6 Å^−1^ and 0.0026–1.4 Å^−1^, respectively. The 2.2–6.7 Å data in between 1.4 and 1.8 Å^−1^ are not flat, which may be caused by the geometric and wavelength effects of the high-angle banks (Karge *et al.*, 2017 ▸). A Debye fit of the scattering profile shows a radius of gyration (*R*_g_) of about 122 Å, corresponding to a molecular weight of 185 kg mol^−1^ according to the scaling between *R*_g_ and molecular weight (Fetters *et al.*, 1994 ▸). The scattering profiles of glassy carbon from SANS2D and VSANS also overlap with each other [Fig. 8 ▸(*c*)] and are about 3% higher than the X-ray calibration of the standard samples (Allen *et al.*, 2017 ▸). PEG-d4 is used to calibrate the time of flight of neutrons from the moderator to the detectors. The peak positions of SANS2D and VSANS coincide with each other [Fig. 8 ▸(*b*)]. Correlation function analysis of the PEG-d4 scattering profiles using *SASView* (https://www.sasview.org/) shows a 17.3 nm lamella thickness of the PEG crystal [Fig. 8 ▸(*d*)] and a better resolution from VSANS than SANS2D, owing to the 12.75 m collimation length of VSANS compared with 4 m for SANS2D. A Bragg peak at 1.67 Å^−1^ can be observed in Fig. 8 ▸(*b*), corresponding to diffraction from the chain (chain distance of about 3.76 Å) inside the crystal layer of the lamella.

**Figure 9:**
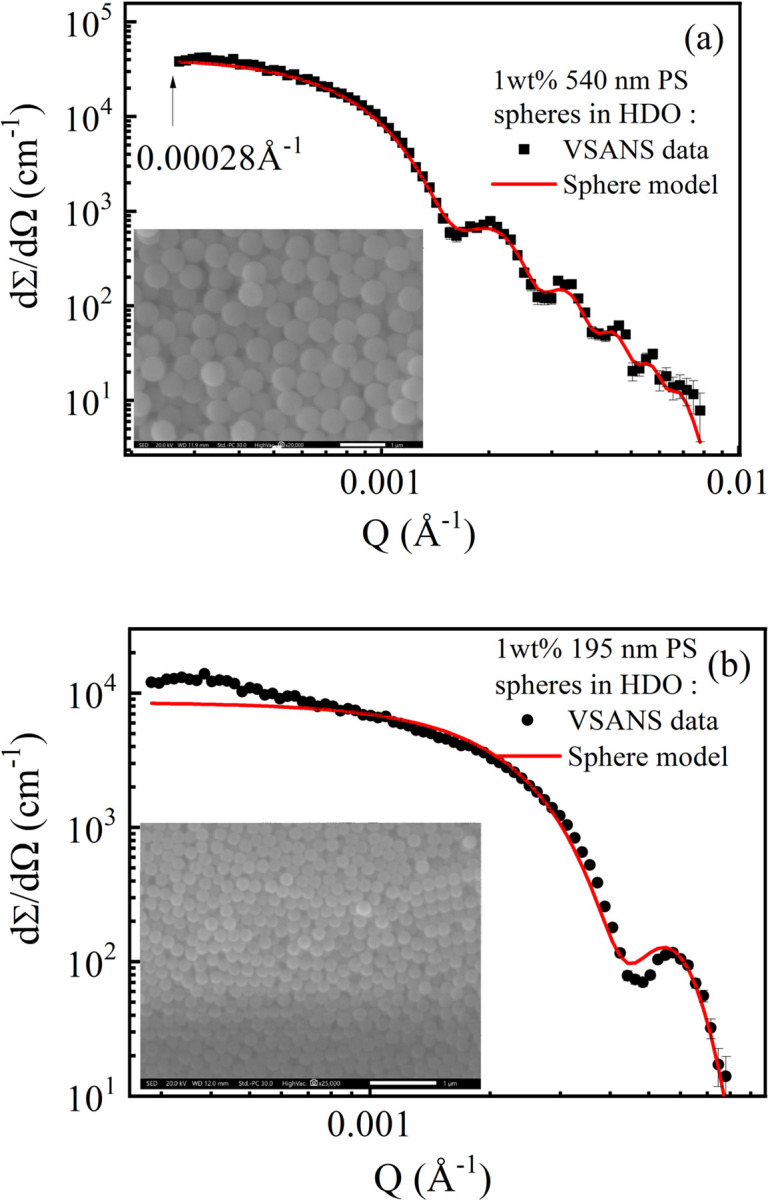
Data from the VSANS mode of CSNS-VSANS compared with the corresponding slit-smeared sphere models: (*a*) 540 nm PS spheres and (*b*) 195 nm PS spheres. The insets are SEM images with scale bars of 1 µm.

The same direct-beam technique was employed to perform the VSANS experiments with two standard samples, 540 and 195 nm diameter PS spheres in DHO at 1 wt% concentration. Data from the two samples were reduced with formula (6) to obtain the scattering profile and absolute scattering intensity. The reduced scattering profiles of the two standard samples were compared with the corresponding slit-smeared (Glatter, 1977 ▸) sphere models (Fig. 9 ▸). The models fit quite well with the data except for a small upturn of the 195 nm sample profile at low *Q*, which may be caused by slight clustering of the particles. The minimum *Q* can reach 0.00028 Å^−1^.

## 5. Conclusion

The CSNS VSANS instrument is designed to be a low-background, flexible and versatile instrument with conventional SANS, multi-slit VSANS and polarizing SANS modes. Key parameters of the instrument are shown in Table 3 ▸. Calibration and initial experiments reveal that all the design goals have been achieved. The featured function of multi-slit focusing pushes the minimum scattering vector to 0.00028 Å^−1^.

A direct-beam technique is employed for the absolute intensity calibration of the instrument. Standard sample measurements show that data from the CSNS VSANS instrument almost overlap with those from a benchmark SANS instrument, validating the effectiveness of the procedure and the instrument’s high performance.

We anticipate that the instrument will deliver new science and discoveries for the scientific community and the industrial world with a broad *Q* coverage and flexibility.

## Figures and Tables

**Table 1:** Parameters of the three disc choppers in normal mode (2.2–6.7 Å) and frame-skipping mode (2.2–11.3 Å)

Choppers	Position (m)	Blade opening angle (°)	Phase of normal mode (°)	Frequency of normal mode (Hz)	Phase of frame-skipping mode (°)	Frequency of frame-skipping mode (Hz)
T1	6.35	73.26	76.16	25	53.24	12.5
T2	8.35	87.74	91.55	25	65.64	12.5
T3	10.9	222.82	232.76	50	168.13	25

**Table 2:** Position and opening and blocking width parameters of the multi-slits

No. of multi-slit	Position from moderator (mm)	Opening width (mm)	Blocking width (mm)	Vertical opening (mm)
1	9250	2.554	1.367	40
2	9940.3	2.485	1.33	40
3	10231.4	2.456	1.314	40
4	10641.5	2.415	1.292	40
5	11213.5	2.358	1.261	40
6	11999.2	2.279	1.219	40
7	13051.9	2.173	1.163	40
8	14408.3	2.038	1.09	40
9	16057.4	1.872	1.002	40
10	17910.7	1.687	0.902	40
11	19806.8	1.497	0.801	40
12	21570	1.32	0.706	40

**Table 3:** Key parameters of the MS-VSANS instrument

Item	Parameters
Source	Coupled hydrogen moderator (CHM 20 K)
Choppers	Three disc choppers
Wavelength band	4.5 Å in normal mode
	9 Å in frame-skipping mode
Source–sample distance	SANS 2.49, 5.15, 9.92 and 12.75 m
	VSANS 12.75 m
Sample–detector distance	SANS 1–11.5 m
	VSANS 12.82 m
Neutron flux at sample	SANS ∼1 × 10^6^ to ∼2 × 10^7^ n s^−1^ cm^−2^ at 100 kW
	VSANS ∼1 × 10^5^ n s^−1^ cm^−2^ at 100 kW
Sample size	SANS ø 6, 8 and 15 mm
	VSANS 15 × 30 mm
Detectors	SANS three ∼1 m^2^ ^3^He PSDs, 8 mm resolution
	VSANS 210 × 210 mm GEM PSD, 2 mm resolution
Typical *Q* ranges	SANS mode 6–10.5 Å, *L*_1_ = 12.75 m: 0.0017–0.6 Å^−1^
	SANS mode 6–10.5 Å, *L*_1_ = 5.15 m: 0.0046–0.6 Å^−1^
	SANS mode 2.2–6.7 Å, *L*_1_ = 12.7 m: 0.0026–1.4 Å^−1^
	SANS mode 2.2–6.7 Å, *L*_1_ = 5.15 m: 0.0068–1.4 Å^−1^
	VSANS mode 6–10.5 Å: 0.00028–0.008 Å^−1^
	VSANS mode 2.2–6.7 Å: 0.00044–0.022 Å^−1^
Polarization	Double-V polarizer having 98% polarization at 2.5 Å
	RF spin flipper
	^3^He spin analyser

## References

[bb1] Allen, A. J., Zhang, F., Kline, R. J., Guthrie, W. F. & Ilavsky, J. (2017). *J. Appl. Cryst.* **50**, 462–474. 10.1107/S1600576717001972 PMC5377342 28381972

[bb2] Barker, J., Moyer, J., Kline, S., Jensen, G., Cook, J., Gagnon, C., Kelley, E., Chabot, J. P., Maliszewskyj, N., Parikh, C., Chen, W., Murphy, R. P. & Glinka, C. (2022). *J. Appl. Cryst.* **55**, 271–283. 10.1107/S1600576722000826 PMC8985601 35497654

[bb3] Carpenter, M. & Faber, J. (1978). *J. Appl. Cryst.* **11**, 464–465.

[bb39] Désert, S., Thévenot, V., Oberdisse, J. & Brûlet, A. (2007). *J. Appl. Cryst.* **40**, s471–s473.

[bb4] Dewhurst, C. D. (2008). *Meas. Sci. Technol.* **19**, 034007.

[bb5] Falcao, A. N., Margaca, F. M. A. & Carvalho, F. G. (2002). *Appl. Phys. Mater. Sci. Process.* **74**, s1462–s1464.

[bb6] Falcão, A. N., Margaça, F. M. A. & Carvalho, F. G. (2003). *J. Appl. Cryst.* **36**, 1266–1269.

[bb7] Fetters, L. J., Hadjichristidis, N., Lindner, J. S. & Mays, J. W. (1994). *J. Phys. Chem. Ref. Data*, **23**, 619–640.

[bb8] Glatter, O. (1977). *J. Appl. Cryst.* **10**, 415–421.

[bb9] Glinka, C. J., Barker, J. G., Hammouda, B., Krueger, S., Moyer, J. J. & Orts, W. J. (1998). *J. Appl. Cryst.* **31**, 430–445.

[bb11] Glinka, C. J., Rowe, J. M. & LaRock, J. G. (1986). *J. Appl. Cryst.* **19**, 427–439.

[bb12] Guo, J., Takeda, S., Morita, S. Y., Hino, M., Oda, T., Kato, J., Yamagata, Y. & Furusaka, M. (2014). *Opt. Express*, **22**, 24666–24677. 10.1364/OE.22.024666 25322041

[bb13] Headen, T. F., Boek, E. S., Stellbrink, J. & Scheven, U. M. (2009). *Langmuir*, **25**, 422–428. 10.1021/la802118m 19063643

[bb14] Heenan, R. K., Penfold, J. & King, S. M. (1997). *J. Appl. Cryst.* **30**, 1140–1147.

[bb15] Heenan, R. K., Rogers, S. E., Turner, D., Terry, A. E., Treadgold, J. & King, S. M. (2011). *Neutron News*, **22**(2), 19–21.

[bb16] Hu, H., Dou, M., Zhang, C., Cheng, H., He, C., Ke, Y., Yuan, B., Bai, B., Sun, Y., Huang, Z., Duan, Y. & Tong, X. (2023*a*). *Rev. Sci. Instrum.* **94**, 074903. 10.1063/5.0138705 37417904

[bb17] Hu, H., Zhang, C., Dou, M., Huang, Z., Sun, Y., Ye, F., Yuan, B., Bai, B., Cheng, H., Yang, S., Duan, Y. & Tong, X. (2023*b*). *Nucl. Instrum. Methods Phys. Res. A*, **1053**, 168317.

[bb18] Huang, C., Zhang, J., Ye, F., Qin, Z., Amir, S. M., Buck, Z. N., Salman, A., Kreuzpaintner, W., Qi, X., Wang, T. & Tong, X. (2021). *Chin. Phys. Lett.* **38**, 092801.

[bb19] Iwase, H., Takata, S., Morikawa, T., Katagiri, M., Birumachi, A. & Suzuki, J. (2018). *Physica B*, **551**, 501–505.

[bb20] Jaksch, S., Martin-Rodriguez, D., Ostermann, A., Jestin, J., Duarte Pinto, S., Bouwman, W. G., Uher, J., Engels, R. & Frielinghaus, H. (2014). *Nucl. Instrum. Methods Phys. Res. A*, **762**, 22–30.

[bb21] Jiang, X.-F., Zhou, J.-R., Luo, H., Xiao, L., Zhou, X.-J., Xu, H., Xia, Y.-G., Wu, X.-G., Zhu, L., Yang, W.-Q., Yang, G.-A., Guan, B.-J., Zhang, H.-Y., Zhao, Y.-B., Sun, Z.-J. & Chen, Y.-B. (2022). *Nucl. Sci. Technol.* **33**, 89.

[bb22] Kampman, N., Busch, A., Bertier, P., Snippe, J., Hangx, S., Pipich, V., Di, Z., Rother, G., Harrington, J. F., Evans, J. P., Maskell, A., Chapman, H. J. & Bickle, M. J. (2016). *Nat. Commun.* **7**, 12268. 10.1038/ncomms12268 PMC4974477 27464840

[bb23] Karge, L., Gilles, R. & Busch, S. (2017). *J. Appl. Cryst.* **50**, 1382–1394. 10.1107/S1600576717011463 PMC5627681 29021734

[bb24] Koizumi, S., Iwase, H., Suzuki, J.-I., Oku, T., Motokawa, R., Sasao, H., Tanaka, H., Yamaguchi, D., Shimizu, H. M. & Hashimoto, T. (2006). *Phys. B Condens. Matter*, **385–386**, 1000–1006.

[bb25] Len, A., Pépy, G. & Rosta, L. (2004). *Physica B*, **350**, E771–E773.

[bb26] Lindner, P. & Schweins, R. (2010). *Neutron News*, **21**(2), 15–18.

[bb27] Liu, D., Khaykovich, B., Gubarev, M. V., Lee Robertson, J., Crow, L., Ramsey, B. D. & Moncton, D. E. (2013). *Nat. Commun.* **4**, 2556. 10.1038/ncomms3556 24077533

[bb28] Luo, T., He, Z. Q., Cheng, H., Zuo, T. S., Xiao, S. W., Lu, S., Ke, Z. Y., Ma, N., Wang, T., Liang, J., Dong, L., Wang, X. L., Li, B. & Men, L. L. (2021). *Nucl. Instrum. Methods Phys. Res. A*, **1010**, 165526.

[bb29] Margaça, F. M. A., Falcão, A. N., Salgado, J. F. & Carvalho, F. G. (2000). *Physica B*, **276–278**, 189–191.

[bb30] Metwalli, E., Götz, K., Lages, S., Bär, C., Zech, T., Noll, D. M., Schuldes, I., Schindler, T., Prihoda, A., Lang, H., Grasser, J., Jacques, M., Didier, L., Cyril, A., Martel, A., Porcar, L. & Unruh, T. (2020). *J. Appl. Cryst.* **53**, 722–733. 10.1107/S1600576720005208 PMC7312133 32684887

[bb31] Mildner, D. F. R. & Carpenter, J. M. (1984). *J. Appl. Cryst.* **17**, 249–256.

[bb32] Nunes, A. C. (1974). *Nucl. Instrum. Methods*, **119**, 291–293.

[bb33] Oku, T., Iwase, H., Shinohara, T., Yamada, S., Hirota, K., Koizumi, S., Suzuki, J.-I., Hashimoto, T. & Shimizu, H. M. (2007). *J. Appl. Cryst.* **40**, s408–s413.

[bb34] Radulescu, A., Kentzinger, E., Stellbrink, J., Dohmen, L., Alefeld, B., Rücker, U., Heiderich, M., Schwahn, D., Brückel, T. & Richter, D. (2005). *Neutron News*, **16**(2), 18–21.

[bb35] Sauli, F. (1997). *Nucl. Instrum. Methods Phys. Res. A*, **386**, 531–534.

[bb36] Sauter, A., Zhang, F., Szekely, N. K., Pipich, V., Sztucki, M. & Schreiber, F. (2016). *J. Phys. Chem. B*, **120**, 5564–5571. 10.1021/acs.jpcb.6b03559 27285548

[bb37] Schmidt, U., Dubbers, D., Raum, K., Joeres, O. & Schärpf, O. (1996). *J. Neutron Res.* **5**, 81–88.

[bb38] Sokolova, A., Whitten, A. E., de Campo, L., Christoforidis, J., Eltobaji, A., Barnes, J., Darmann, F. & Berry, A. (2019). *J. Appl. Cryst.* **52**, 1–12.

[bb40] Thiyagarajan, P., Epperson, J. E., Crawford, R. K., Carpenter, J. M., Klippert, T. E. & Wozniak, D. G. (1997). *J. Appl. Cryst.* **30**, 280–293.

[bb41] Vogtt, K., Siebenbürger, M., Clemens, D., Rabe, C., Lindner, P., Russina, M., Fromme, M., Mezei, F. & Ballauff, M. (2014). *J. Appl. Cryst.* **47**, 237–244.

[bb42] Wang, F., Liang, T., Yin, W., Yu, Q., He, L., Tao, J., Zhu, T., Jia, X. & Zhang, S. (2013). *Sci. China Phys. Mech. Astron.* **56**, 2410–2424.

[bb43] Wang, Z., Wu, H., Chen, L., Sun, L. & Wang, X. (2018). *J. Appl. Cryst.* **51**, 1605–1615.

[bb44] Wignall, G. D. & Bates, F. S. (1987). *J. Appl. Cryst.* **20**, 28–40.

[bb45] Yamada, M., Iwashita, Y., Ichikawa, M., Fuwa, Y., Tongu, H., Shimizu, H. M., Mishima, K., Yamada, N. L., Hirota, K., Otake, Y., Seki, Y., Yamagata, Y., Hino, M., Kitaguchi, M., Garbe, U., Kennedy, S. J., Tung Lee, W., Andersen, K. H., Guerard, B., Manzin, G. & Geltenbort, P. (2015). *Prog. Theor. Exp. Phys.* **2015**, 043G01.

[bb46] Yamada, M., Iwashita, Y., Kanaya, T., Yamada, N. L., Shimizu, H. M., Mishima, K., Hino, M., Kitaguchi, M., Hirota, K., Geltenbort, P., Guerard, B., Manzin, G., Andersen, K., Lal, J., Carpenter, J. M., Bleuel, M. & Kennedy, S. J. (2011). *Physica B*, **406**, 2453–2457.

[bb47] Zhang, H., Cheng, H., Yuan, G., Han, C. C., Zhang, L., Li, T., Wang, H., Liu, Y. T. & Chen, D. (2014). *Nucl. Instrum. Methods Phys. Res. A*, **735**, 490–495.

[bb48] Zhang, J., Huang, C., Qin, Z., Ye, F., Amir, S. M., Salman, A., Dong, Y., Tian, L., Buck, Z. N., Kreuzpaintner, W., Musgrave, M., Qi, X., Wang, T. & Tong, X. (2022). *Sci. China Phys. Mech. Astron.* **65**, 241011.

[bb49] Zhao, J. K., Gao, C. Y. & Liu, D. (2010). *J. Appl. Cryst.* **43**, 1068–1077.

[bb50] Zhou, J., Zhou, X., Zhou, J., Jiang, X., Yang, J., Zhu, L., Yang, W., Yang, T., Xu, H., Xia, Y., Yang, G., Xie, Y., Huang, C., Hu, B., Sun, Z. & Chen, Y. (2020*a*). *Nucl. Eng. Technol.* **52**, 1277–1281.

[bb51] Zhou, J., Zhou, X., Zhou, J., Teng, H., Yang, J., Ma, Y., Zhou, K., Xia, Y., Xiu, Q., Yang, T., Jiang, X., Zhu, L., Yang, W., Yang, G., Xie, Y., Hu, B., Sun, Z. & Chen, Y. (2020*b*). *Nucl. Instrum. Methods Phys. Res. A*, **962**, 163593.

[bb52] Zhou, Z., Bouwman, W. G., Schut, H., Desert, S., Jestin, J., Hartmann, S. & Pappas, C. (2016). *Carbon*, **96**, 541–547.

[bb53] Zuo, T., Lu, Z., Deng, C., Xiao, S., He, Y., He, Z., Lin, X., Ma, C., Han, Z. & Cheng, H. (2022). *Quantum Beam Sci.* **6**, 25.

[bb54] Zuo, T. S., Cheng, H., Chen, Y. B. & Wang, F. W. (2016). *Chin. Phys. C*, **40**, 076204.

